# Separase Cleaves the N-Tail of the CENP-A Related Protein CPAR-1 at the Meiosis I Metaphase-Anaphase Transition in *C*. *elegans*

**DOI:** 10.1371/journal.pone.0125382

**Published:** 2015-04-28

**Authors:** Joost Monen, Neil Hattersley, Andrew Muroyama, Deanna Stevens, Karen Oegema, Arshad Desai

**Affiliations:** Ludwig Institute for Cancer Research & Department of Cellular and Molecular Medicine, University of California San Diego, La Jolla, California, United States of America; University of Connecticut, Storrs, UNITED STATES

## Abstract

Centromeres are defined epigenetically in the majority of eukaryotes by the presence of chromatin containing the centromeric histone H3 variant CENP-A. Most species have a single gene encoding a centromeric histone variant whereas *C*. *elegans* has two: HCP-3 (also known as CeCENP-A) and CPAR-1. Prior RNAi replacement experiments showed that HCP-3 is the functionally dominant isoform, consistent with CPAR-1 not being detectable in embryos. GFP::CPAR-1 is loaded onto meiotic chromosomes in diakinesis and is enriched on bivalents until meiosis I. Here we show that GFP::CPAR-1 signal loss from chromosomes precisely coincides with homolog segregation during anaphase I. This loss of GFP::CPAR-1 signal reflects proteolytic cleavage between GFP and the histone fold of CPAR-1, as CPAR-1::GFP, in which GFP is fused to the C-terminus of CPAR-1, does not exhibit any loss of GFP signal. A focused candidate screen implicated separase, the protease that initiates anaphase by cleaving the kleisin subunit of cohesin, in this cleavage reaction. Examination of the N-terminal tail sequence of CPAR-1 revealed a putative separase cleavage site and mutation of the signature residues in this site eliminated the cleavage reaction, as visualized by retention of GFP::CPAR-1 signal on separating homologous chromosomes at the metaphase-anaphase transition of meiosis I. Neither cleaved nor uncleavable CPAR-1 were centromere-localized in mitosis and instead localized throughout chromatin, indicating that centromere activity has not been retained in CPAR-1. Although the functions of CPAR-1 and of its separase-dependent cleavage remain to be elucidated, this effort reveals a new substrate of separase and provides an *in vivo* biosensor to monitor separase activity at the onset of meiosis I anaphase.

## Introduction

Centromeres direct chromosome segregation by building kinetochores, the protein machines that form dynamic attachments to spindle microtubules and function as scaffolds for signaling pathways that ensure accuracy in chromosome segregation [1,2]. In most eukaryotes, centromeres are not defined by the underlying DNA sequence but are instead defined by the presence of chromatin containing the specialized histone H3 variant called CENP-A [3–5]. The mechanisms that ensure propagation of CENP-A nucleosomal chromatin across cell division and the features of this specialized chromatin that direct kinetochore assembly are both areas of active investigation [6].

While many species have CENP-A chromatin restricted to a specific region of the chromosome (the centromere), there are also a large number of extant species in distinct lineages where CENP-A chromatin is more broadly distributed throughout the genome [7–11]. In nematode and plant holocentric species, CENP-A chromatin coalesces along the entire outer surface of each chromatid to form a platform for assembly of a diffuse kinetochore [7,9–12]. Recent work suggests that insect holocentric species have altogether lost CENP-A and instead build their kinetochores by an alternative mechanism [13]. The best experimentally studied holocentric species to date is *C*. *elegans*, a popular model organism in biological research. One unusual feature of *C*. *elegans* is that, unlike the majority of eukaryotes, it harbors two CENP-A related proteins: HCP-3 (also referred to as CeCENP-A) and CPAR-1. Prior work has shown that HCP-3 is the dominant isoform and is required for recruitment of all kinetochore proteins and thus for accurate segregation of chromosomes during embryonic cell divisions [8,14,15]. Surprisingly, HCP-3 is not required for meiotic kinetochore formation or chromosome segregation [16,17]. The functional importance of CPAR-1 is not understood except that it is highly enriched on meiotic chromosomes and is not detectable in embryos. Here we analyze the dynamics of CPAR-1 during the transition from oocyte meiosis to embryonic mitoses. GFP::CPAR-1 signal is abruptly lost from chromosomes coincident with anaphase onset of meiosis I. We show that this signal loss likely reflects direct cleavage within the N-terminal tail of CPAR-1 by the protease separase. Both cleaved CPAR-1 and an uncleavable mutant of CPAR-1 are not centromere-localized in embryos, indicating that CPAR-1 has lost centromere activity. Although the functional significance of CPAR-1 cleavage by separase is currently unclear, these results reveal a new substrate for separase and provide a biosensor for precisely timing separase activation during meiosis I.

## Results & Discussion

### Centromeric histone-encoding gene duplication events in *Caenorhabditis* species

**Fig 1:**
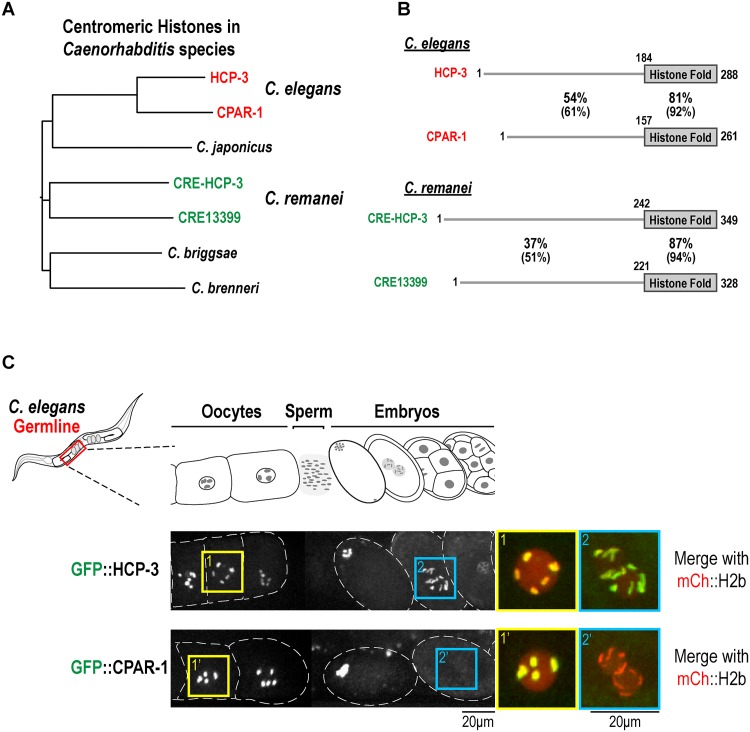
Duplicated CENP-A related genes in Caenorhabditis species. **(A)** Tree generated by primary sequence alignments of CENP-A related proteins in the indicated *Caenorhabditis* species. The sequences were obtained from Wormbase [40]. Alignments were performed using Muscle [44] implemented in Jalview 2 [45]. The tree was constructed in the Clustal W Phylogeny tool [46], employing the neighbor-joining method and default parameters. The alignment was imported into FigTree v1.3.1 [47] for formatting and export. **(B)** Primary sequence features of the two CENP-A related proteins in *C*. *elegans* and *C*. *remanei*. In all 4 proteins an N-terminal tail (N-tail), significantly longer than the N-tail of canonical histone H3 or human Cenp A, is followed by a histone fold domain (HFD). The tail & histone fold alignments were done using Blast and percent identity and similarity (in brackets) is reported; gaps are not reported. See also Fig 4A. HCP-3 and CPAR-1 comparison adapted from *Monen et al*. [16]. **(C)** Images of adult *C*. *elegans* worms expressing single copy GFP transgene insertions of HCP-3 (OD421) and CPAR-1 (OD416) under their endogenous 5’ and 3’ UTR. GFP was fused to the N-terminus of each CENP-A related protein [7]; the GFP::HCP-3-expressing transgene was crossed into an *hcp-3Δ* mutant, which it fully rescues. The region of the germline where oocytes are fertilized, pass through the spermatheca and begin early embryogenesis is shown. The boxed regions magnified on the right are of oocyte chromosomes (box 1, 1’) and of prometaphase one-cell embryo chromosomes (box 2, 2’). Scale bars are 20 μm; blowups are magnified an additional 2-fold.

The centromeric histone H3 variant is generally encoded by a single gene, including invertebrate and fungal species that arose following whole genome duplications [18,19]. The presence of HCP-3 and CPAR-1 in *C*. *elegans* is therefore somewhat unusual. The high primary sequence homology (Fig 1B [16]) and intronic nucleotide sequence homology between *hcp-3* and *cpar-1* genomic loci suggests that this duplication is relatively recent. To assess if this duplication was unique to *C*. *elegans*, we searched the sequenced and annotated genomes of four other *Caenorhabditis* species: *C*. *briggsae*, *C*. *brenneri*, *C*. *japonica*, and *C*. *remanei*. The *C*. *remanei* genome also harbors two genes encoding CENP-A related proteins (Fig 1A and 1B). Primary sequence alignments indicate that independent duplication events are responsible for the presence of two genes encoding CENP-A related proteins in *C*. *elegans* and *C*. *remanei* (Fig 1A and 1B). Thus, the gene encoding the centromeric histone variant has been duplicated at least twice in *Caenorhabditis* species.

### GFP::CPAR-1 is lost from chromosomes coincident with oocyte meiosis I anaphase

As reported previously, GFP::HCP-3 expressed from a targeted single copy transgene insertion rescues a deletion mutant of *hcp-3* and localizes to chromosomes in both meiotic oocytes and in developing embryos (Fig 1C [7,20]). By contrast, GFP::CPAR-1 expressed under control of its endogenous promoter from a targeted single copy transgene insertion is restricted to meiotic oocytes and is not observed in mitotically dividing embryos (Fig 1C [20]). This restricted pattern suggests that CPAR-1 is removed from chromosomes between meiosis and mitosis.

As fertilized oocytes transition from meiosis to mitosis, a number of proteins are degraded by a mechanism under control of the kinase MBK-2 [21–23]. A prominent substrate of this degradation mechanism is the microtubule severing protein MEI-1/katanin, which is essential for acentrosomal spindle assembly in meiosis I and II, but is detrimental to centrosomal spindle assembly in the embryo [24–26]. MEI-1 and other oocyte-to-embryo transition-associated targets are degraded following meiosis II [27].

**Fig 2:**
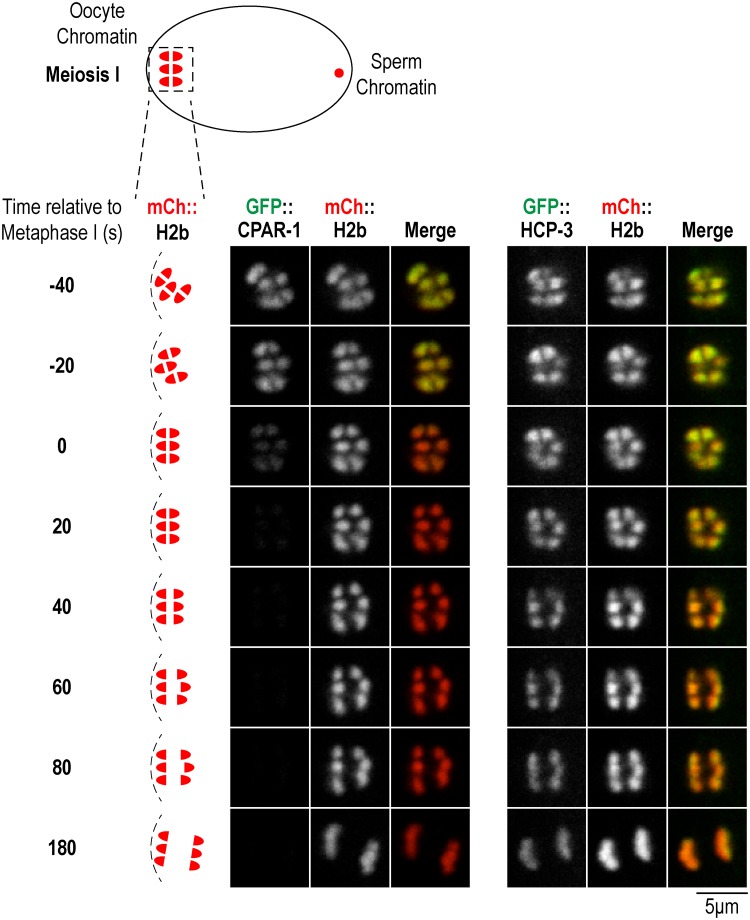
GFP::CPAR-1 signal is lost from chromosomes at the metaphase-anaphase transition of oocyte meiosis I. Image frames from time-lapse sequences of GFP::CPAR-1 (OD416) and GFP::HCP-3 (OD421) meiosis I embryos (single copy insertions as in Fig 1). Chromosomes were labeled with mCherry::H2b. In the GFP::HCP-3 strain, the GFP fusion is the sole source of HCP-3. The images shown are from prometaphase through late anaphase. Metaphase is defined as the first frame in which chromosomes are aligned and spindle is rotated to be perpendicular to cortex. Schematics on the left indicate the stages shown. Similar results were obtained for n = 10 time-lapse sequences. Scale bar, 5 μm.

To assess if CPAR-1 may be a substrate of this pathway, we monitored the timing of GFP::CPAR-1 signal loss, and in parallel analyzed GFP::HCP-3. Both GFP::CPAR-1 and GFP::HCP-3 were detected on bivalent chromosomes throughout chromosome alignment (Fig 2, S1 and S2 Movies). GFP::HCP-3 signal continued to persist on chromosomes during anaphase segregation (S1 Movie). By contrast, GFP::CPAR-1 signal was abruptly lost at the meiosis I metaphase-anaphase transition (Fig 2 and S2 Movie). Consistent with the observed timing of signal loss, no GFP::CPAR-1 signal was observed on chromosomes during meiosis II or embryonic mitotic divisions (Fig 1C). Thus, the timing of the GFP signal loss argues against GFP::CPAR-1 being a target of the degradation pathway operating at the oocyte-to-embryo transition, and instead suggests a linkage to the meiosis I metaphase-to-anaphase transition.

### CPAR-1::GFP, unlike GFP::CPAR-1, is retained on segregating anaphase chromosomes during meiosis I

**Fig 3:**
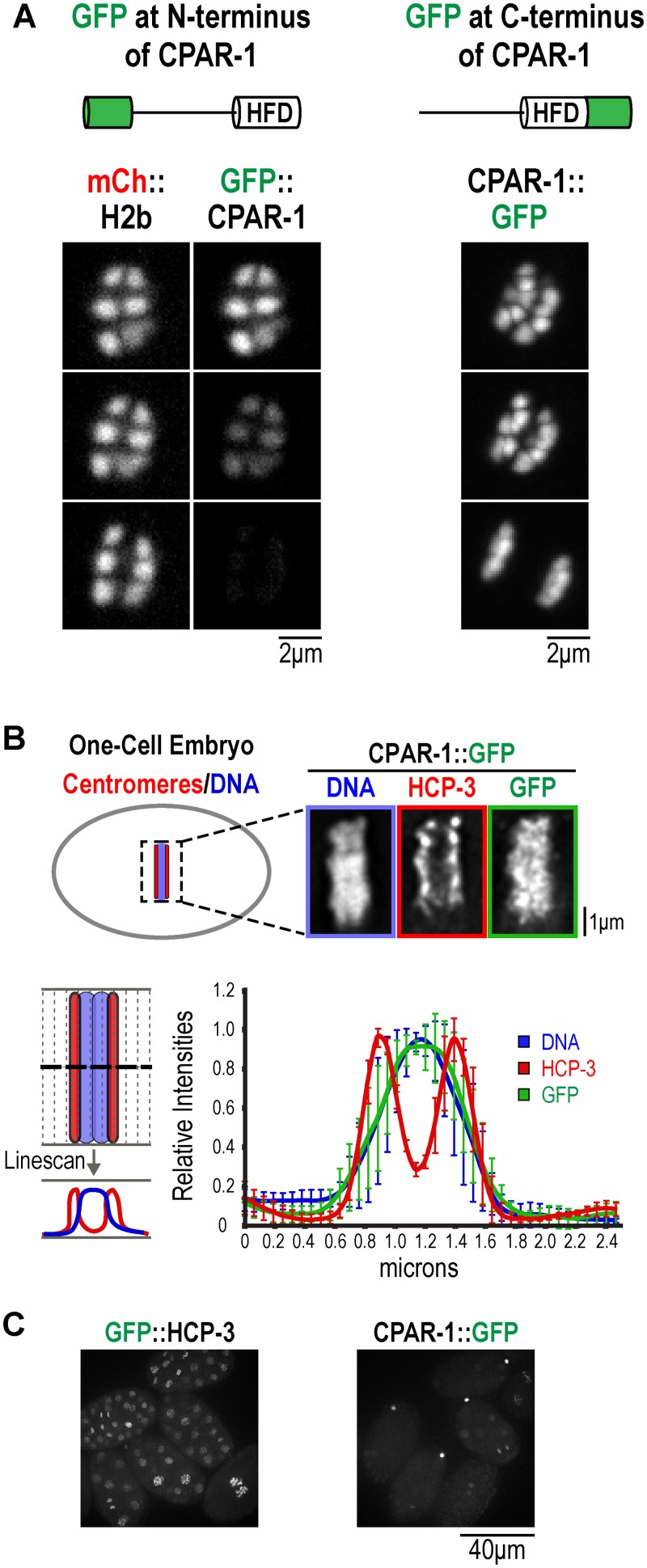
The GFP signal of CPAR-1::GFP is retained on meiotic chromosomes but is not centromeric in mitosis. **(A)** Comparison of GFP::CPAR-1 (OD82) to CPAR-1::GFP (OD145) in meiosis I anaphase. HFD refers to the histone fold domain of CPAR-1. The GFP signal is abruptly lost at meiosis I anaphase onset for GFP::CPAR-1 but is unchanged for CPAR-1::GFP. Similar results were observed for n = 12 time-lapse sequences. Scale bars, 2 μm. **(B)** Immunofluorescence analysis of one-cell mitotic embryos expressing CPAR-1::GFP (OD145). Embryos were fixed and stained for DNA, HCP-3, and GFP, to detect the cleaved CPAR-1::GFP. A representative image of a metaphase embryo is shown. Scale bar, 1 μm. Linescan analysis on 6 embryos was performed using a 47-pixel wide line drawn as depicted. Linescans were aligned using peak Hoechst intensity as the midline, normalized and the averaged profiles plotted. Error bars are the standard deviations. **(C)** Comparison of GFP::HCP-3 (OD421) to CPAR-1::GFP (OD145) in multi-cellular mitotic embryos. GFP::HCP-3 exhibits robust nuclear localization in all cells while CPAR-1::GFP is very weakly detected in embryos, with prominent signal in polar bodies. The images were collected with equal exposure and illumination intensity and processed identically after acquisition.

The abrupt loss of GFP::CPAR-1 signal could reflect either removal of CPAR-1 from chromosomes or a proteolytic event that releases the GFP moiety from chromatin. To distinguish between these two possibilities, we generated *pie-1* promoter-driven transgenes expressing GFP::CPAR-1 (OD82) or CPAR-1::GFP (OD145)—where GFP was fused to the C-terminus of CPAR-1, distal to the histone fold rather than to the N-terminal tail—and integrated them into the genome using biolistic transformation [28]. Similar to the endogenous promoter-driven GFP::CPAR-1 (Fig 2), the *pie-1* promoter-driven GFP::CPAR-1 signal was lost at the metaphase-anaphase transition of meiosis I. CPAR-1::GFP localized similarly to GFP::CPAR-1 on bivalent chromosomes in oocytes and in prometaphase and metaphase of meiosis I (Fig 3A and S3 Movie). However, no loss of GFP signal from chromosomes was observed for CPAR-1::GFP (Fig 3A and S3 Movie). Thus, the loss of GFP signal at the onset of anaphase of meiosis I observed for GFP::CPAR-1 is not due to its removal from chromatin but likely due to a cleavage event between the GFP and the histone fold of CPAR-1 that liberates GFP from chromatin. Due to the inability to detect CPAR-1 by immunoblotting [16], we were unable to obtain biochemical evidence for cleavage by comparing mobility of CPAR-1 in meiosis I metaphase-arrested versus mitotic embryos.

To determine if cleaved CPAR-1 retains centromere activity, we fixed and stained one-cell CPAR-1::GFP expressing embryos for HCP-3, GFP (to visualize the cleaved CPAR-1::GFP), and DNA (Fig 3B). Both qualitative images and linescan analysis of condensed chromosomes indicated that, unlike HCP-3 which was restricted to the characteristic ‘two-stripe’ pattern of diffuse kinetochores on these holocentric chromosomes, the cleaved CPAR-1::GFP was not centromeric and instead localized throughout chromosomal DNA (Fig 3B). Additionally, in multi-cellular embryos CPAR1::GFP was not significantly detected in nuclei except transiently on chromosomes in mitosis, unlike the prominent localization of GFP::HCP-3 throughout embryogenesis (Fig 3C).

### Separase inhibition prevents loss of GFP::CPAR-1 signal from chromosomes in meiosis I

**Fig 4:**
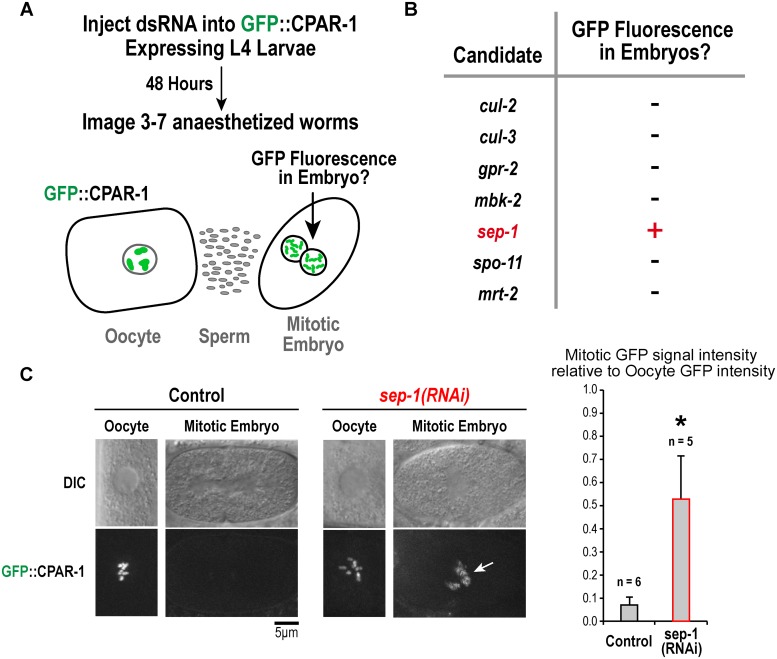
A small candidate screen implicates separase in the loss of chromosomal GFP::CPAR-1 signal observed at meiosis I anaphase onset. **(A)** Schematic of the experimental strategy employed to screen candidate genes for their role in the loss of chromosomal GFP::CPAR-1 signal. **(B)** Summary of the results for the tested candidate genes. Only depletion of separase/SEP-1 resulted in presence of GFP signal on chromosomes of mitotic embryos, indicating that the normal cleavage-associated loss of GFP::CPAR-1 from chromosomes at meiosis I anaphase did not occur. **(C)** Example images of GFP::CPAR-1 (OD82) on oocyte and embryo chromosomes in control and *sep-1(RNAi)* worms. GFP::CPAR-1 intensities in oocytes and embryos were quantified by taking average projections of 10 μm thick sections (6 slices total) in a 5 μm by 5 μm region of interest encompassing the chromosomes. Background signal intensity was subtracted out of each projected stack and integrated GFP signal intensities were normalized relative to the average value in oocytes. GFP fluorescence was reduced to 0.070 +/- 0.035 (n = 6) in embryos compared to oocytes in controls; in contrast, for *sep-1(RNAi)* GFP fluorescence was 0.528 +/- 0.185 (n = 5) in embryos compared to oocytes (p < 0.0001 relative to control). Arrow points to retention of GFP signal on embryo chromosomes in *sep-1(RNAi)*. Scale bar, 5 μm.

The retention of GFP signal for CPAR-1::GFP, in contrast to the complete loss of GFP signal for GFP::CPAR-1, suggested that CPAR-1 undergoes proteolytic cleavage in its N-terminal tail at the onset of meiosis I anaphase. To test this idea, we conducted a small candidate screen to assess the requirements for loss of GFP::CPAR-1 signal at this stage (Fig 4A). Tested candidates included proteins involved in the meiosis-to-mitosis transition (*mbk-2*, *cul-2*, *cul-3*) and in cleavage of the kleisin subunit of the cohesin complex (*sep-1*); as controls, we also analyzed components involved in meiotic recombination (*mrt-2* and *spo-11*), and cortical force generation (*gpr-2*). In brief, we depleted worms expressing *pie-1* promoter-driven GFP::CPAR-1 (OD82) and assessed if GFP signal was detected on mitotic chromosomes of embryos, suggesting that the normal meiosis I anaphase-associated cleavage event did not occur. Of the 7 tested genes, only depletion of separase (SEP-1) affected the dynamics of GFP::CPAR-1 (Fig 4B and 4C). Compared to control worms, in which GFP::CPAR-1 signal was observed in oocytes but not in embryos (Fig 4C), *sep-1(RNAi)* worms exhibited GFP::CPAR-1 signal in one-cell mitotic embryos (Fig 4C). *sep-1(RNAi)* oocytes fail meiotic chromosome segregation but continue cell cycle progression and enter the mitotic program. Separase inhibition was confirmed by the presence of extra chromosomes in mitotic embryos and the lack of polar bodies, which is a hallmark of failed meiotic chromosome segregation; eggshell formation was also defective (*not shown* [29]). These data implicate separase in the loss of GFP::CPAR-1 signal on meiotic chromosomes at meiosis I anaphase onset.

### Mutation of a predicted separase cleavage site in the CPAR-1 N-tail prevents loss of GFP::CPAR-1 signal from chromosomes at meiosis I anaphase

A role for separase in GFP::CPAR-1 cleavage is consistent with its activation at anaphase onset during both meiosis and mitosis. Separase is recruited to chromosomes and proteolytically cleaves the kleisin subunit of the cohesin complex (in *C*. *elegans*, the kleisin subunits are REC-8 and COH-3/4 in meiosis, SCC-1 in mitosis), releasing homologues (in meiosis I) or sister chromatids (meiosis II and mitosis) [30–34].

**Fig 5:**
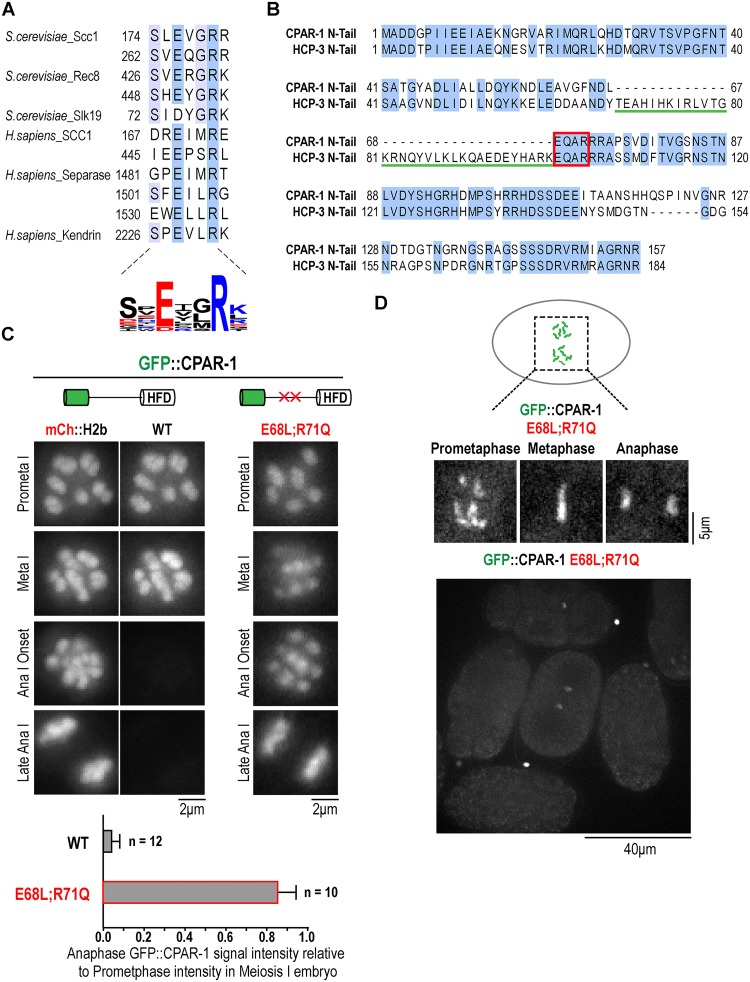
Mutation of a predicted separase cleavage site in the CPAR-1 N-terminal tail prevents loss of GFP::CPAR-1 from chromosome at the meiosis I metaphase-anaphase transition. **(A)** Summary of known separase cleavage motifs in the kleisin subunits of the cohesin complex, in the fungal spindle protein Slk19, in vertebrate separase, and in the pericentriolar material protein kendrin. Blue shading indicates conservation of amino acid residues. Sequence alignment performed using Muscle [44] in Jalview 2 [45]. A sequence logo summarizing the distribution of amino acids in the cleavage sites is shown below. Color indicates amino acid charge. Sequence logo was generated with Weblogo (http://weblogo.threeplusone.com/create.cgi) [48,49]. **(B)** Alignment of the N-terminal tails of HCP-3 and CPAR-1. Both N-tails harbor an EQAR motif that resembles a separase cleavage site. Note the 33 amino acid sequence immediately upstream of this motif in HCP-3 that is absent in CPAR-1. **(C)** Image frames from time-lapse sequences of meiosis I embryos expressing either GFP::CPAR-1^WT^ (OD416) or GFP::CPAR-1^E68L;R71Q^ (OD180). GFP::CPAR-1 intensities were quantified by taking average projections of 8 μm thick sections (5 slices total) in a 5 μm by 5 μm region of interest encompassing the chromosomes. Background signal intensity was subtracted out of each projected stack and integrated GFP signal was normalized relative to average integrated GFP signal on chromosomes in prometaphase I. For WT GFP::CPAR-1, mCherry::H2b was crossed in and imaged at the same time to highlight the abrupt loss of GFP fluorescence. No loss of GFP fluorescence was observed at meiosis I anaphase onset for the E68L;R71Q mutant form of GFP::CPAR-1. GFP signal loss from chromosomes was quantified on chromosomes 60 seconds post anaphase I onset relative to chromosomes at prometaphase I within the same cell. For GFP::CPAR-1, chromosomal GFP signal dropped 60 seconds after the metaphase-to-anaphase transition to 0.04 +/- 0.05 (n = 12), relative to prometaphase I. In contrast, for GFP::CPAR-1^E68L;R71Q^, chromosomal GFP signal persisted at 0.85 +/- 0.09 (n = 10), relative to prometaphase I. Scale bars, 2 μm. * p-value < 0.0001. **(D)** Frames from a time-lapse sequence of a one-cell mitotic embryo expressing GFP::CPAR-1^E68L;R71Q^ (OD180). Similar results were observed in n = 3 embryos. Scale bar, 5 μm. Lower panel shows a field of multi-cellular embryos expressing GFP::CPAR-1^E68L;R71Q^.

Two mechanisms may explain the separase-dependent loss of GFP::CPAR-1 signal at the metaphase-anaphase transition in meiosis I. First, CPAR-1 may be a direct substrate for separase cleavage, analogous to the kleisin subunits of cohesin. Alternatively, separase may indirectly trigger CPAR-1 cleavage through its action on another substrate. Pioneering work in budding yeast identified a motif required for separase cleavage, E-x-x-R, in the mitotic and meiotic kleisins and in the fungal-specific spindle-associated protein Slk19 (Fig 5A [35,36]). Two additional separase substrates have been described in metazoans: separase itself, which undergoes autocleavage in vertebrates but not in fungi [37,38], and the pericentriolar material protein kendrin [39]. Thus, only three direct separase substrates, other than kleisins, have been identified to date. The E-x-x-R motif appears to be conserved in all of the known substrates (Fig 5A) and mutation of the key glutamic acid and arginine residues to leucine and glutamine, respectively, abrogates cleavage [36].

Analysis of the amino acid sequences of the N-terminal tails of CPAR-1 and HCP-3 revealed a putative separase cleavage motif (E-Q-A-R) in both (Fig 5B). CPAR-1 lacks a 33 amino acid insertion that immediately precedes the separase cleavage motif in HCP-3 and is predicted to be alpha helical based on secondary structure predictions. To test whether the ‘EQAR’ motif was required for separase-dependent loss of GFP::CPAR-1 signal during meiosis I, we introduced mutations previously described to abolish separase cleavage [36]. We generated a *pie-1* promoter-driven transgene expressing GFP::CPAR-1^E68L;R71Q^ and integrated it into the genome using biolistic transformation [28]. In contrast to GFP::CPAR-1 expressing embryos, in embryos expressing GFP::CPAR-1^E68L;R71Q^, GFP signal was retained on chromosomes without any significant loss of fluorescence during the metaphase-anaphase transition of meiosis I (Fig 5C and S4 Movie). This result strongly suggests that direct separase cleavage of the EQAR motif in the N-tail of CPAR-1 is the cause of the loss of GFP::CPAR-1 signal. We note that the EQAR sequence is conserved in HCP-3, which does not appear to be cleaved by separase to the same degree as CPAR-1 (Fig 2). We speculate that this difference could be due to two reasons. First, based on systematic biochemical analysis of budding yeast separase cleavage specificity [36], the residues upstream of the EQAR motif in HCP-3, most notably the arginine residue 2 amino acids upstream of the glutamate in the consensus sequence, make it a poor substrate for cleavage compared to CPAR-1. Second, the insertion immediately upstream of the cleavage motif in HCP-3 may either modify the potential for the EQAR motif to be a substrate or block access of the cleavage site.

Finally, we analyzed the localization of uncleavable GFP::CPAR-1^E68L;R71Q^ in mitotic one-cell embryos to assess if CPAR-1 has retained centromere activity. Similar to the cleaved CPAR-1 (Fig 3B), the uncleavable GFP::CPAR-1^E68L;R71Q^ mutant did not exhibit the characteristic 2-striped pattern observed for HCP-3 and instead resembled chromosomal DNA (Fig 5D). Thus, neither cleaved nor uncleavable CPAR-1 exhibits centromeric activity in mitosis.

## Conclusions

Here we provide evidence that CPAR-1, a recent gene duplication-derived CENP-A related protein in *C*. *elegans*, is cleaved in its N-terminal tail by separase at the meiosis I metaphase-anaphase transition. CPAR-1 cannot be detected by immunoblotting [16], presumably because of its highly restricted expression, which has precluded obtaining biochemical evidence for the cleavage. In addition, neither active *C*. *elegans* separase nor *C*. *elegans* centromeric histone-containing nucleosomes have been reconstituted, both of which will be necessary to demonstrate direct cleavage. Thus, biochemical evidence for direct cleavage of the CPAR-1 N-tail by separase remains to be obtained. Nevertheless, the evidence for this cleavage event occurring and being due to separase directly acting on the CPAR-1 N-tail is strong. First, the timing of GFP signal loss for a single copy insertion of GFP::CPAR-1, expressed from it’s endogenous UTRs, precisely coincides with anaphase onset, when separase is activated. Second, loss of GFP signal from chromatin depends on the placement of the GFP tag—when GFP is placed distal to the histone fold, no fluorescence loss is observed indicating that signal loss is due to GFP being liberated from the chromatinized histone fold. Third, separase inhibition prevents signal loss of GFP::CPAR-1. Fourth, and most compellingly, mutation of two residues in a putative separase cleavage site in the N-tail abolishes GFP::CPAR-1 signal loss. Collectively, these observations provide strong support for the claim that separase is directly cleaving CPAR-1 in its N-terminal tail. Thus, GFP::CPAR-1 is an effective sensor for separase activation in meiosis I.

The two major questions raised by the observations reported here are: 1) what is the function of CPAR-1, and 2) what is the function of separase-mediated cleavage of the CPAR-1 N-tail. Unfortunately, despite significant effort we have been unable to address these questions to date. The only available mutant allele that disrupts the *cpar-1* locus is *tm2612* [40]. In this allele, a significant portion of the *cpar-1* locus, which closely abuts the *emb-30* gene encoding an essential subunit of the APC/C, is deleted. A homozygous *tm2612* mutant is zygotic lethal (i.e. heterozygous *tm2612* mothers lay 25% inviable homozygous *tm2612* embryos), suggesting an essential function in the deleted region. However, RNAi-mediated selective depletion of CPAR-1 (performed by employing a strain where an HCP-3 transgene is recoded to be RNAi-resistant and the dsRNA injected targets both endogenous HCP-3 and CPAR-1 due to their high nucleotide sequence homology) does not lead to embryonic lethality [7]. In addition, we have been unable to rescue the *tm2612* allele using either tagged or untagged *cpar-1* transgenes that include extended upstream and downstream control regions, making it an open question as to whether the lethality observed in *tm2612* is due to deletion of the *cpar-1* locus or due to another reason. We have also attempted to test if the lethality in *tm2612* is due to perturbation of the adjacent *emb-30* gene, by generating an *emb-30* transgene insertion, but this effort also failed to demonstrate rescue. The inability to rescue *tm2612* has precluded our efforts to understand the functions of CPAR-1 and the significance of the separase-mediated cleavage in its N-terminal tail at the onset of meiosis I anaphase. Generation of new *cpar-1* deletion mutants is needed to address these two major questions raised by our observations.

## Materials and Methods

### Live imaging, GFP fusions, and site-directed mutagenesis

All live images were acquired via either a spinning disk confocal microscope (CSU10; McBain Instruments) mounted on an inverted microscope (TE2000e; Nikon) using a 60× 1.4 NA plan Apo objective with 1.5× auxiliary magnification and a cooled CCD camera (Orca ER; Hamamatsu) binning 2 × 2, spinning disk confocal microscope (CSU10—Yokogawa Corporation of America) mounted on an inverted microscope (TE2000-E; Nikon) equipped with a100x 1.4 NA Plan Apochromat lens (Nikon), solid-state 100-mW lasers, and a high resolution EMCCD iXon camera (Andor Technology; 1 × 1 binning), or a DeltaVision-modified inverted microscope (IX70; Olympus) and Softworx software (Applied Precision) using 2×2 binning with a 100× NA 1.3 U-planApo objective. To construct strain OD82 (strain containing *pie-1* promoter driven N-terminally tagged GFP:CPAR-1^CeCENP-A^), oligonucleotides CGCTTCCACTAGTGCCGATGACGGACCAATTAT and CGCTTCCACTAGTTCAGAGATTTGGAAGGCAAAG were used to PCR CPAR-1 from cDNA, and the resulting fragment was digested with SpeI and cloned into pIC26 (S1 Table). The resulting plasmid (pOD248/pPM12) was biolistically transformed into *C*. *elegans* to generate a stable integrated strain. A similar approach was used to create the CPAR-1::GFP strain (OD145) with the only difference being the CPAR-1 PCR product was cloned into pAZ132 resulting in a pJM19 plasmid containing c-terminally tagged CPAR-1 (S1 Table). To create the worm strain expressing the mutated *pie-1* promoter driven N-terminally tagged GFP::CPAR-1^E68L_R71Q^, site directed mutagenesis was performed on pOD248 using QuickChange II XL (Agilent Technologies) with oligonucleotides GCTGTTGGATTCAACGACTTACTACAAGCTCAAAGAAGAGCTCCTTCTGTGG and CCACAGAAGGAGCTCTTCTTTGAGCTTGTAGTAAGTCGTTGAATCCAACAGC to create pJM65, which was subsequently biolistically transformed into *C*. *elegans* to generate the stable integrated strain OD180 (S1 Table). The single copy insertion GFP::HCP-3 and GFP::CPAR-1 worm strains (OD421 and OD416 respectively) were generated as described (S1 Table) [7]. GFP::CPAR-1 intensities in oocytes and embryos were quantified by taking average projections of 10 μm thick sections (6 slices total) in a 5 μm by 5 μm region of interest encompassing the chromosomes. Background signal intensity was subtracted out of each projected stack and overall GFP signal intensities were normalized to average intensity levels in oocytes (Fig 4C) or average intensity levels of chromosomes in pro-metaphase I (Fig 5C).

### Fixation, immunofluorescence, and line-scan analysis

Embryos were fixed by freeze cracking and plunging into -20°C methanol as described [41]. Fixation times were 2 h and embryos were rehydrated in PBS, blocked in AbDil (PBS plus 2% BSA, 0.1% Triton X-100), incubated overnight at 4°C with 1 μg/ml of each directly labeled antibodies against amino-acids 2–143 of HCP-3 and GFP monoclonal diluted in AbDil, washed with PBST (PBS plus 0.1% Triton X-100), incubated for 1 h with FITC anti—mouse secondary (Dianova GmbH), washed with PBST, with PBST plus 1 μg /ml Hoechst, and mounted in 0.5% p-phenylenediamine, 20 mM Tris-Cl, pH 8.8, 90% glycerol. Three- dimensional widefield datasets were collected using a Deltavision microscope at 1×1 binning with a 100× NA 1.3 U-planApo objective. The images were computationally de-convolved using softWoRx (Applied Precision) and maximum intensity projections were imported into Adobe Photoshop CS5 (Adobe) for further processing. Using softWoRx (Applied Precision), horizontal line-scans of vertically positioned individual chromosomes in prometaphase and metaphase were quantified to compare signal intensity levels between antibody and Hoechst localization.

### Candidate gene screen and RNAi

RNAi was performed by injection of dsRNA against the target gene as described previously [42]. Injected worms were incubated at 20°C for 48 h prior to imaging. dsRNA Injected whole worms were anesthetized in M9 media containing 0.1% tricane (Sigma, St. Louis, MO), mounted on agarose pads and imaged at 20°C on a spinning disk confocal (Eclipse TE2000-E; Nikon, Melville, NY) microscope equipped with a Nikon 60×, 1.4NA PlanApo oil objective lens and a Hamamatsu ORCA-ER CCD camera. GFP::CPAR-1 intensities in oocytes and embryos were quantified by taking average projections of 10 μm thick sections (6 slices total) in a 5 μm by 5 μm region of interest encompassing the chromosomes. Background signal intensity was subtracted out of each projected stack and overall GFP signal intensities were normalized to average intensity levels in oocytes using ImageJ software [43]. The oligonucleotides used to generate dsRNAs for the screen are as follows:

*mbk-2*, AATTAACCCTCACTAAAGGCACGATCATATTGCGTACCG and TAATACGACTCACTATAGGTCGTCACCCATGTTCTTCAA;
*cul-2*, AATTAACCCTCACTAAAGGCGAGAATGTACCCCAGCAAT and TAATACGACTCACTATAGGGCAGATAGTCACCGCTCACA;
*cul-3*, AATTAACCCTCACTAAAGGAGCGTGCCATACAGGAAATC and TAATACGACTCACTATAGGCTTAAGCAGCGCCTTCAATC;
*gpr-2*, AATTAACCCTCACTAAAGGAGAGTGTGCAGGCATTTGAA and TAATACGACTCACTATAGGACGGGTTCCTCGTTCTCTTT;
*mrt-2*, TAATACGACTCACTATAGGATGATGGAATTAGAAACGGGTCA and AATTAACCCTCACTAAAGGCTTGATAATCTCCTTGAGAACTT;
*spo-11*, AATTAACCCTCACTAAAGGGGGATAATTCGTGGAGCAAT and TAATACGACTCACTATAGGCGACAGCGAACATTGTGAAT;
*sep-1*, TAATACGACTCACTATAGGAATCATTCCATCCGATCACC and AATTAACCCTCACTAAAGGTTCCGTCGTTTCCAAAACTC. Each of the dsRNAs created utilized standard methods as previously described [42].

## Supporting Information

S1 Movie Time-lapse video of meiosis I in oocyte expressing GFP::HCP-3. The time-lapse video captures the metaphase-to-anaphase transition in meiosis I, starting at prometaphase I and ending at anaphase I in a GFP::HCP-3 (OD421) expressing oocyte. GFP signal is retained on chromatin after the metaphase-to-anaphase transition. The video was processed as a 5 z-stack maximum projection, 8 μm (2 μm spacing) thick. Frames were captured at 20-second intervals and replayed at 7 frames per second (140x real-time). The embryos are oriented anterior left. Chromosomes on the left will form the first polar body and chromosomes on the right will continue into meiosis II. The scale of the video is 13.1 x 13.1 μm.
(MOV)

S2 Movie Time-lapse video of meiosis I in oocyte expressing mCherry:: Histone-H2B and GFP::CPAR-1. The time-lapse video captures the metaphase-to-anaphase transition in meiosis I, starting at prometaphase I and ending at anaphase I in a GFP::CPAR-1;mCherry::H2b (OD416) expressing oocyte. mCherry signal is on the left and GFP signal is on the right. The time-lapse shows abrupt removal of GFP from chromatin at anaphase I onset. The video was processed as a 5 z-stack maximum projection, 8 μm (2 μm spacing) thick. Frames were captured at 20-second intervals and replayed at 7 frames per second (140x real-time). The embryos are oriented anterior left. Chromosomes on the left will form the first polar body and chromosomes on the right will continue into meiosis II. The scale for each channel is 13.1 x 13.1 μm.
(MOV)

S3 Movie Time-lapse video of meiosis I in oocyte expressing CPAR-1::GFP. The time-lapse video captures the metaphase-to-anaphase transition in meiosis I, starting at prometaphase I and ending at anaphase I in a CPAR-1::GFP (OD145) expressing oocyte. GFP signal is retained on chromatin after the metaphase-to-anaphase transition. The video was processed as a 5 z-stack maximum projection, 8 μm (2 μm spacing) thick. Frames were captured at 20-second intervals and replayed at 7 frames per second (140x real-time). The embryos are oriented anterior left. Chromosomes on the left will form the first polar body and chromosomes on the right will continue into meiosis II. The scale of the video is 13.1 x 13.1 μm.
(MOV)

S4 Movie Time-lapse video of meiosis I in oocyte expressing GFP::CPAR-1^E68L;R71Q^. The time-lapse video captures the metaphase-to-anaphase transition in meiosis I, starting at prometaphase I and ending at anaphase I in a GFP::CPAR-1^E68L;R71Q^ (OD180) expressing oocyte. GFP signal is retained on chromatin after the metaphase-to-anaphase transition. The video was processed as a 5 z-stack maximum projection, 8 μm (2 μm spacing) thick. Frames were captured at 20-second intervals and replayed at 7 frames per second (140x real-time). The embryos are oriented anterior left. Chromosomes on the left will form the first polar body and chromosomes on the right will continue into meiosis II. The scale of the video is 13.1 x 13.1 μm.
(MOV)

S1 Table *C*. *elegans* strains used in this study. (XLSX)
